# Enumerating the forest before the trees: The time courses of estimation-based and individuation-based numerical processing

**DOI:** 10.3758/s13414-020-02137-5

**Published:** 2020-09-30

**Authors:** David Melcher, Christoph Huber-Huber, Andreas Wutz

**Affiliations:** 1 Center for Mind/Brain Sciences and Department of Psychology and Cognitive Sciences, University of Trento, Corso Bettini 31, 38068 Rovereto, Italy; 2 Psychology Program, Division of Science, New York University Abu Dhabi, Abu Dhabi, UAE; 3 Donders Institute for Brain, Cognition and Behaviour, Radboud University, Nijmegen, Netherlands; 4 Center for Cognitive Neuroscience, University of Salzburg, Salzburg, Austria; 5 Picower Institute for Learning and Memory, MIT, Cambridge, MA USA

**Keywords:** Object recognition, Scene perception, Temporal processing

## Abstract

Ensemble perception refers to the ability to report attributes of a group of objects, rather than focusing on only one or a few individuals. An everyday example of ensemble perception is the ability to estimate the numerosity of a large number of items. The time course of ensemble processing, including that of numerical estimation, remains a matter of debate, with some studies arguing for rapid, “preattentive” processing and other studies suggesting that ensemble perception improves with longer presentation durations. We used a forward-simultaneous masking procedure that effectively controls stimulus durations to directly measure the temporal dynamics of ensemble estimation and compared it with more precise enumeration of individual objects. Our main finding was that object individuation within the subitizing range (one to four items) took about 100–150 ms to reach its typical capacity limits, whereas estimation (six or more items) showed a temporal resolution of 50 ms or less. Estimation accuracy did not improve over time. Instead, there was an increasing tendency, with longer effective durations, to underestimate the number of targets for larger set sizes (11–35 items). Overall, the time course of enumeration for one or a few single items was dramatically different from that of estimating numerosity of six or more items. These results are consistent with the idea that the temporal resolution of ensemble processing may be as rapid as, or even faster than, individuation of individual items, and support a basic distinction between the mechanisms underlying exact enumeration of small sets (one to four items) from estimation.

Visual scenes are typically crowded and contain numerous objects. An example is a set table for a large dinner party, which might contain a series of plates, glasses, and silverware. Such a scene can be perceived in terms of individual objects, such as by fixating on a particular glass in order to grasp it. However, we are also able to quickly and effectively glean the overall meaning of the table and report the average color, size, and shape of the plates or glasses and give a close, but often inexact, estimate of the number of place settings.

Participants are able to report the exact combination and properties of features for an attended object, and even the exact feature value for a small group of objects (Cowan, 2000; Xu & Chun, 2009). The representation of features for a group of items, however, reflects an estimation of the average value of the item’s orientation, size, motion, color, or even more complex features such as gender or facial expression (for review, see Whitney & Yamanashi Leib, 2018). This estimation of the “feature gist” of the scene, or average value of an ensemble of items, may help to provide the rich impression of continuous and stable perception over time and across saccades (Corbett, Fischer, & Whitney, 2011; Corbett & Melcher, 2014a, 2014b; Melcher & Colby, 2008).

One of the attributes of ensemble perception that has often been emphasized is its speed. It has been suggested that the properties of the ensemble, such as average size, shape, or orientation, are perceived in a single glance (for a dissenting viewpoint, however, see Myczek & Simons, 2008). A stronger claim, however, is that “ensemble representations can be extracted with a temporal resolution at or beyond the temporal resolution of individual object recognition” (Whitney & Yamanashi Leib, 2018, p. 112). There is evidence for extraction of ensemble information from very brief presentations or from rapid sequences (Haberman & Whitney, 2007). For example, Chong and Treisman (2003) reported that mean size could be computed for a display shown for only 50 ms, which is consistent with the idea of rapid, parallel feature processing (see also Yamanashi Leib et al., 2016). That study, however, did not mask the stimuli, so the effective duration of the display was likely much longer. When using a backward mask, Whiting and Oriet (2011) found that size averaging improved for durations greater than 100 ms, suggesting that an effective duration of 100–200 ms was required in their task to reach maximal performance. When interpreting the time course of mental operations, like estimation, it is critical to distinguish between their temporal resolution, which defines how quickly sufficient information can be obtained to make a reasonable judgment about the stimulus, and their temporal integration window, which defines at which point performance does not improve significantly with increased viewing time (Whitney & Yamanashi Leib, 2018).

In terms of our understanding of visual processing, the question of whether features can be extracted into an ensemble within 50 ms, or rather 200 ms, is critical theoretically. More generally, processing of ensembles or scene “gist” is often claimed to be faster than object recognition (amongst others; Greene & Oliva, 2009a, 2009b), which would be a requirement for any theory that posits the use of scene context to disambiguate object identity (e.g., Bar, 2004). To directly compare ensemble perception to processing of individual objects, then, requires measuring both the temporal resolution (“can ensemble processing be done for a presentation rate of 50 ms?”) and temporal integration window (“does precision improve over longer time periods?”) of both processes.

The comparison of ensembles versus individual perception is further complicated by the fact that even the perception of individual objects might vary depending on the number of items in question (Wutz, Caramazza, & Melcher, 2012; Wutz & Melcher, 2013, 2014; Wutz, Weisz, Braun, & Melcher, 2014). Perhaps the best-known demonstration of rapid object individuation is the classic Sperling (1960) study showing that participants can report only around four items in a single glance with near perfect accuracy. In terms of rapid encoding for enumeration (“how many items are there?”) or visual memory (“was this item presented previously?”) tasks, many studies have shown that performance remains high up to around three to four items, depending on the stimulus and task parameters and individual differences. This raises the additional question of whether the appropriate comparison for ensemble processing is processing a single item or, instead, that of individuating a small group of items.

Our ability to either focus on an individual item or, instead, the group as a whole is captured by the saying of “not seeing the forest for the trees” (attributed to Sir Thomas More, 1533). Here, we are asking whether we actually do see the forest before the trees, as measured by our ability to report the number of trees. To do so, we compare the time course of seeing the numerosity of “the forest” with that of perceiving exactly one, two, three or more “trees”—that is, we compare the time course of ensemble processing with that of individual item perception in the case of rapid enumeration.

## Estimation versus individuation

Individuation is a core mental ability. It requires the perceptual system to select features from an image, bind them into an object, and select it as separate from other objects and from the background (Xu & Chun, 2009). Individuation forms a primary constraint for the limited capacity of perception, attention, and working memory (Piazza, Fumarola, Chinello, & Melcher, 2011; Wutz & Melcher, 2013). Indeed, when looking at single participants, it has been found that individuation commonly exceeds working memory capacity and that the two measures can be highly correlated (Melcher & Piazza, 2011; Piazza et al., 2011). Moreover, the stages of individuation and then identification in scene analysis are clearly dissociable by different masking techniques (Wutz & Melcher, 2013). This body of evidence suggests that individuation forms a critical limit for mental capacity, upon which higher cognitive tasks like working memory or object-location tracking are grounded (Dempere-Marco, Melcher, & Deco, 2012), with the additional requirements of identification for working memory or location updating for multiple-object tracking.

In addition to its central role for scene analysis and object capacity, individuation is fundamental for numerical cognition. The phenomenon of “subitizing” reflects the rapid apprehension of the numerosity of a small set of items (Jevons, 1871; Kaufman, Lord, Reese, & Volkmann, 1949). Typically, around three to four items can be individuated at once (as described above) and can be enumerated precisely and exactly, whereas quantities exceeding this limit are only represented by approximation (estimation of the numerosity of the ensemble) or involve multiple processing steps (counting).

For numerical cognition, it has been argued that individuation and estimation are distinct processes operating on distinct object quantities (Burr, Turi, & Anobile, 2010; Feigenson, Dehaene, & Spelke, 2004; Piazza et al., 2011). For example, numerosity judgments typically follow Weber’s law, with errors increasing in proportion to the number of items presented, while errors remain relatively constant within the subitizing range (Burr et al., 2010). Moreover for scene analysis, estimation of the quantity of items in an ensemble is considered central, because it allows for the global processing of image properties that gives access to the large-scale scene layout and its summary statistics (Alvarez, 2011; Alvarez & Oliva, 2008, 2009). In this view, estimation may be a special case of ensemble or statistical processing, key to our sense of number and in some ways similar to ensemble processing of other basic visual features, such as orientation, size, movement, color, and depth, as well as more complex features such as the gender or emotional expression of a face (for review, see Whitney & Yamanashi Leib, 2018).

## The time course of estimation and individuation

In terms of individuation, capacity limits have typically been characterized in terms of space, forming a debate between theories involving a finite number of discrete slots and those that argue for limited shared resources among the processed items (Awh, Barton, & Vogel, 2007; Cowan, 2000; Xu & Chun, 2009). Such theories start with the idea of how many items in space are individuated (and encoded into working memory) in a single glance. However, more recent accounts that investigated individuation with highly time-sensitive measures (e.g., visual masking, magnetoencephalography) support an alternative explanation based on time (Wutz et al., 2012; Wutz & Melcher, 2013, 2014; Wutz et al., 2014). The pattern of results in those studies suggests that individuation is not instantaneous, and that time may be a key factor that plays a role in capacity limits. Those studies divided the effective processing of stimuli (or, depending on interpretation, the iconic or sensory memory) into smaller units of time by means of forward masking (see below and Method section). In line with classic psychophysical estimates of sensory memory, individuation capacity limits (of around three or four items) were reached only when the effective duration of the targets was at least 100–150 ms. One or two items, by contrast, could be accurately individuated quite quickly after ca. 30–50 ms. That pattern of results suggests that individuation capacity depends on the temporal limits imposed by the fading trace of sensory memory. It is important to note that mere stimulus detection did not depend on temporal factors, in the same paradigm, suggesting that individuation operates on a subsequent level of scene analysis beyond simple feature detection (Wutz & Melcher, 2013).

## The current study

In the present study, we now directly compared how individuation and estimation evolve over time within the same individuals using the above-described forward masking paradigm. Typically, performance in both individuation and estimation tasks has been measured in terms of a single display presentation, with little control over the effective duration of the stimuli in terms of visual persistence and sensory memory. Here, instead, we take advantage of a masking paradigm that combines simultaneous and forward masking (Di Lollo, 1980). This method involves a forward pattern mask upon which, at some stimulus onset asynchrony (SOA), one or more targets (“X” symbols) are superimposed.

**Fig. 1:**
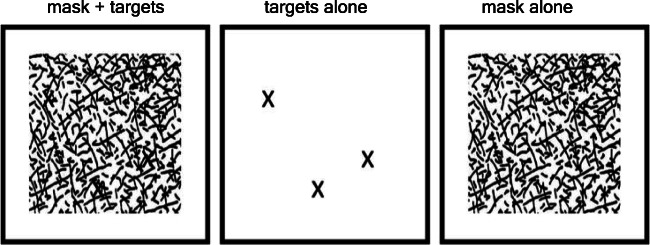
Illustration of the stimulus. The simultaneous mask plus target display is shown in the leftmost panel. The two component parts of the mask plus target display are shown in the middle panel (the X-shaped targets) and the right panel (the mask pattern of oriented lines). The X-shaped targets and the mask pattern were superimposed together in the mask plus target display presented on each trial. The mask plus target display (left panel) always contained the mask and at least one X-shaped target. The mask display (right panel) always contained only the pattern of oriented lines without any X-shaped targets. By presenting first the mask display and then the mask plus target display (made up of both the Xs and the same mask pattern) with sufficient stimulus onset asynchrony (SOA), the Xs appearing on top of the mask pattern became visible. In contrast, when there is no SOA (or when it is very brief), the Xs are either not visible or difficult to exactly enumerate. On each trial, the mask pattern of oriented lines was unique and was matched for that trial in both the target and the mask displays. Note that the X-shaped targets were never presented alone, but always superimposed on top of the mask pattern of oriented lines. Further details regarding the stimulus parameters and the order of events on each trial can be found in the Method section

One key aspect of this design is simultaneous masking. As shown in Fig. 1, the target is camouflaged by the mask, since both are made up of black lines. It is impossible to detect the targets when they are presented together with the mask at an SOA of zero ms. When the SOA exceeds around 20–30 ms, it is possible to detect that there is at least one target (Di Lollo, 1980; Wutz et al., 2012). However, for greater quantities within the subitizing range (three to four items), exact enumeration performance is only reached at longer SOAs of around 100–150 ms (Wutz et al., 2012; Wutz & Melcher, 2013, 2014; Wutz et al., 2014). In other words, the ability to distinguish between different item quantities at different set sizes depends on the SOA between the first display (only mask) and second display (targets and mask together).

The paradigm was designed to yield a form of integration pattern masking due to the close temporal proximity between the two displays. Integration masking describes the temporal aspect of the paradigm, in which both displays (mask and target displays) are combined into a single unified percept. When there is only a single, unique percept, the target Xs are not visible. Instead, when the display is temporally segregated into two unique events, the target Xs become visible (for review, see Enns & Di Lollo, 2000). Pattern (or “structure”) masking describes the fact that the two displays (mask and target) contain similar component elements, which here are oriented lines (for review, see Enns & Di Lollo, 2000). The main reason for using the combined forward and simultaneous (pattern) masking paradigm is that it is provides greater control over the effective processing time of the multiple targets, because very short SOAs can be used (Wutz et al., 2012). By contrast, traditional backward masking paradigms typically leave time for the stimulus presentation duration before the mask is presented. Thus, classic forward or backward masking yields more of an all-or-none step function in which the targets are either visible, and thus enumerated correctly within the subitizing range, or invisible (Wutz et al., 2012; Wutz & Melcher, 2013). For object individuation, we expected to replicate our earlier findings that performance within the subitizing range increases up to capacity limits within 100–150 ms. Moreover, we mapped out the time course of object-quantity *estimation* and compared it with the temporal dynamics of *individuation*. Similar time courses would suggest a common underlying mechanism for individuation and estimation and would therefore raise a critical argument against theories that involve extraction of ensemble representations (or scene “gist”) prior to object identification. Alternatively, one process might be faster/slower suggesting that the one builds upon the other or that they serve complementary visual strategies. In particular, if feature processing is rapid and parallel, this could serve estimation and drive a fast temporal resolution for estimation that could even exceed that of the exact individuation of objects.

## Experiment 1

### Method

#### Participants

Eighteen participants (10 females, 17 right-handed, mean age = 24.4 years, *SD* = 1.9 years) took part in the experiment. All participants provided written informed consent, as approved by the institutional ethics committee. They took part in exchange for course credit or a small payment and had normal or corrected-to-normal vision. This sample size (*N* = 18) is similar to our previous work with the same paradigm (*N* = 14 in Wutz et al., 2012; *N* = 16 in Wutz & Melcher, 2013; *N* = 14 in Wutz et al., 2014). In our earlier studies, we found strong main effects for SOA (η_p_^2^ ~ 0.8–0.9) and for item numerosity (η_p_^2^ ~ 0.5–0.7), and small-sized to medium-sized interaction effects (η_p_^2^ ~ 0.1–0.3). Based on a power analysis (using the software G*Power; Faul, Erdfelder, Lang, & Buchner, 2007), the minimum required sample size to detect small-sized to medium-sized effects (*f* = 0.2) is 16 participants (analysis of variance [ANOVA], repeated measures, within factors, 16 measurements, alpha = 0.05, power = 0.8). Thus, our sample (*N* = 18) is sufficient to detect the expected pattern of results.

#### Stimuli and apparatus

**Fig. 2:**
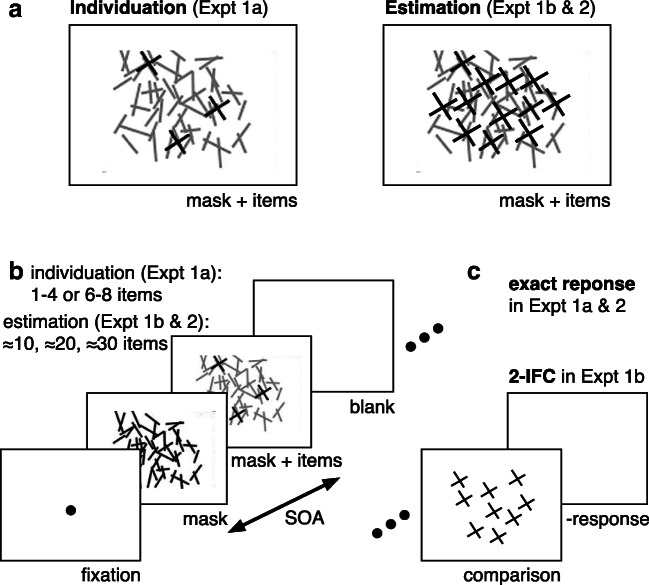
Stimuli and trial design. **a** Mask plus target display with few items for the individuation task (Expt 1a, left) and many items for the estimation task (Expt 1b and 2, right). The mask is shown in grey in the “mask plus items” display for illustrative purposes only. In the experiment, all lines were presented at the same (black) level of contrast in both displays. The size of the targets and mask is shown for illustrative purposes only and does not match the actual displays (see Fig. 1 for a more realistic illustration of the mask plus target displays). **b** A typical trial in the experiment. The masking procedure is identical for individuation (Expt 1a) and estimation (Expt 1b and 2). **c** The tasks only differ in the response procedure (via number key for reporting an exact number for individuation in Expt 1a and for estimation in Expt 2, or with two-interval forced choice, 2-IFC, for estimation in Expt 1b)

The experiment was run using MATLAB (The MathWorks, Natick, MA) and Psychophysics Toolbox (Version 3; Brainard, 1997; Pelli, 1997). Participants were seated in a dimly lit room, approximately 45 cm from a CRT monitor (1,280 × 1,024 resolution, 36.5 × 27.2-cm display size) running at 60 Hz. On each trial a different pattern of 1,080 randomly oriented, partially crossing black lines (mean line length = 1° visual angle [VA], mean line width = 0.1° VA, 31 × 25° VA mean size of whole pattern) was presented centered on a white background (see Fig. 2a). This pattern remained on the screen, and then, after a variable onset delay, a variable number of items (depending on each task estimation or individuation) appeared. The target items formed an “X” and were linearly superposed upon the random line pattern, by use of the image processing technique “alpha blending”—that is, using Screen(‘BlendFunction’) in Psychophysics Toolbox (Version 3; Brainard, 1997; Pelli, 1997). A small set size (one to eight items) was used for individuation in Experiment (Expt) 1a, and a large set size (>10 items) was used for estimation in Expts 1b and 2 (see Figs. 1 and 2a). The physical properties of both mask and target elements (i.e., contrast, mean line length, mean line width) were equated and the alpha-blending procedure edited the transparency/opacity values of the visual stimuli assuring a mathematically correct superimposition, such that all of the lines were the same level of black. All items were colored in black, were 1° VA in size, and were placed randomly on one of 100 possible locations within an invisible, central rectangle of 14.5° VA in eccentricity (horizontally and vertically) with a minimum buffer of 0.5° VA between the locations.

#### Procedure

In the first experiment, each subject completed the two tasks (individuation in Expt 1a, estimation in Expt 1b) in two sessions comprising approximately 1 hour each. The serial order of the tasks was balanced across the observers. All subjects received verbal and written instructions about each task and completed 20 practice trials prior to the main experiment. Each trial began with a central fixation dot (black, 0.5° VA) on a white background for 500 ms. Then, the random line masking pattern (containing oriented black lines) was presented for a specific duration to control the stimulus onset asynchrony (SOA) between the onset of the mask and the target item(s), which were made up of the black lines from the mask plus the black “X” symbols. For estimation (Expt 1b) there were five SOAs (33, 50, 100, 200, 500 ms) and one “no-mask” control condition. We used the same four SOAs between 33–200 ms for individuation (Expt 1a), because this process has been shown previously to unfold within this temporal range (Wutz et al., 2012; Wutz & Melcher, 2013). We focused on this common SOA range for the two tasks for the subsequent analysis. The target display containing the items to be individuated or estimated was superposed upon the masking pattern and was always presented for the same brief duration of 50 ms. The mask plus target display was immediately followed by a white screen (see Fig. 1b). Using this procedure, we achieved an optimal temporal resolution to slice visual processing time (sensory/iconic memory) after target exposure into small units of only tens of milliseconds, as very short SOAs can be used. Short SOAs leave less time to exclusively access the target item(s) memory trace, because the sensory traces of the mask and the target item(s) are temporally integrated for a greater amount of time (Di Lollo, 1980; Wutz et al., 2012; Wutz & Melcher, 2013).

The quantity of the target item(s) and the response procedure after mask plus target item(s) presentation depended on the specific task (see Fig. 1c). For individuation in Expt 1a, we either showed items within the subitizing range (one to four items) or a randomly assigned quantity between six and eight items serving as a control condition outside the subitizing range. We informed the participants that quantities in the range between one and eight items would be shown. Participants did not know that a five-item target was never presented. They were instructed to respond their perceived item quantity by pressing the corresponding number on a keyboard immediately after the presentation of the mask plus target display.

For estimation in Expt 1b, randomly assigned item quantities in three different number ranges were used. Either 11–15 (thereafter called ≈10 items), 21–25 (≈20 items) or 31–35 items (≈30 items) were shown on each trial. The response procedure followed a two-interval forced-choice design (see Fig. 2c). The white screen after the mask-plus-item(s) presentation remained on the screen for 1 s, and then a second item display without a mask was shown for 200 ms, against which the target item(s) had to be compared. A white screen immediately followed the presentation of the second item display. The comparison quantity was a constant Weber fraction above/below the sample quantity (±3–5 items for 11–15 items, ±6–10 items for 21–25 items, ±9–15 items for 31–35 items), to control for difficulty across number ranges. Participants were instructed to press the left arrow key when they perceived the sample as containing more elements than the comparison and the right arrow key otherwise. For both tasks, the subject’s response on the keyboard initiated the next trial.

The individuation experiment (Expt 1a) comprised 10 blocks of 80 trials each. Each combination of mask item(s) SOA (33, 50, 100, 200 ms) and item number (one to four items or “above-capacity” control) was shown four times per block in random order. The estimation experiment (Expt 1b) comprised 10 blocks of 72 trials each. For five subjects, only eight blocks were available due to technical difficulties. Each combination of mask item(s) SOA (33, 50, 100, 200, 500 ms or no mask control) and item number (≈10, ≈20, ≈30 items) was shown four times per block in random order. The sample contained more items than the comparison display on half of the trials and less on the remaining half.

#### Data analysis

The performance (percentage correct trials) for each task (individuation, estimation) was fed into a two-way repeated-measures analysis of variance (ANOVA), with the factors time (i.e., SOA) and number range. For individuation only data for number ranges within the subitizing range (one to four items) was analyzed. Because the 500 ms SOA and the “no-mask” condition were not present for the individuation task, they were left out for the analysis for the estimation task. Performance in the 500 ms SOA condition was very similar to the other SOAs (500 ms SOA: *M* = 79.5% correct, *SD* = 5.7% correct; other SOAs: *M* = 79.5% correct, *SD* = 4.9% correct). The “no-mask” condition yielded slightly worse performance (*M* = 74.2% correct, *SD* = 8% correct), probably because it was a much less frequent event than the masked conditions throughout the block. To directly compare the performance across tasks, we averaged over the number ranges in each task (one to four items for individuation, ≈10–≈30 items for estimation) and ran a two-way repeated-measures ANOVA, with the factors time (i.e., SOA from 33–200 ms) and task (individuation, estimation). We used partial eta square (η_p_^2^) to calculate effect sizes for the ANOVA results. Further, we measured the individuation performance increase with longer SOA by means of logarithmic function fits with two free parameters (y = a × log (b × x)). The logarithmic fits were used to estimate the SOA-value at which performance was at a critical threshold of 75%-correct responses, for each numerosity level separately.

### Results

#### Individuation over time and number (Expt 1a)

**Fig. 3:**
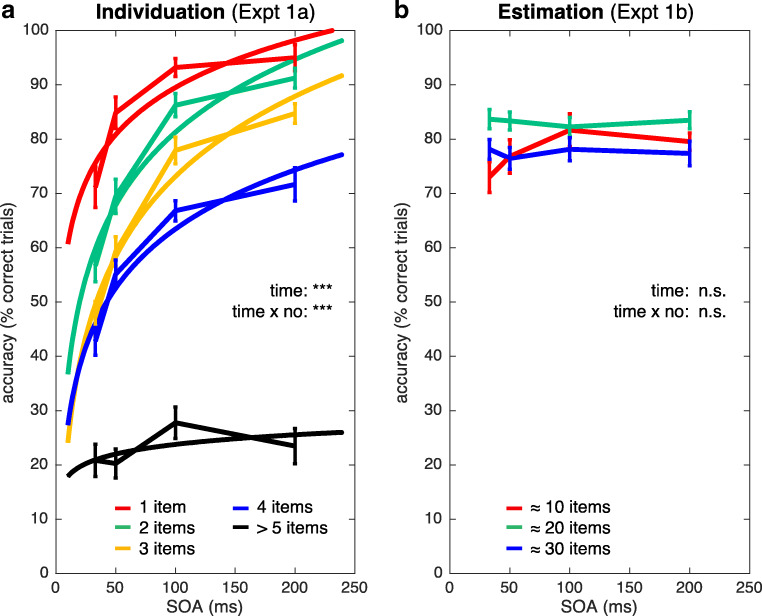
Performance per task. **a** Performance (% correct trials) and logarithmic function fits for the individuation task as a function of SOA and number. **b** Performance (% correct trials) for the estimation task as a function of SOA and number. Error bars reflect the standard error of the mean for repeated-measures designs (Morey, 2008). The central insets show the ANOVA results (n.s. = not significant; ***p < .001)

We found significant main effects of both item number (within the subitizing range, one to four items) and time (33–200 ms SOA) on individuation performance, number: *F*(3, 51) = 51.3, *p* < 2 × 10^-15^, η_p_^2^ = 0.75; time: *F*(3, 51) = 60.7, *p* < 1.1 × 10^-16^, η_p_^2^ = 0.78. Moreover, both factors showed a significant interaction, *F*(9, 153) = 3.4, *p* < 7.4 × 10^-4^, η_p_^2^ = 0.17 (see Fig. 3a). This pattern of results is remarkable, because typically individuation performance is at ceiling and indistinguishable for item numbers within the subitizing range. The masking procedure used here, however, revealed strong differences even between one and four items. Moreover, individuation performance increased with increasing SOA between mask and target item display. This suggests that individuation is not an instantaneous process but instead depends on temporal factors. Moreover, the interaction pattern confirms our earlier findings (Wutz et al., 2012; Wutz & Melcher, 2013) that the masking procedure affects the rate at which items are individuated. Individuation capacity increased in steps with increasing time left in visual processing/memory. It is important to note that individuation performance outside the subitizing range showed a qualitatively and quantitatively different pattern. As expected, performance for 6-8 items was considerably worse compared with smaller numerosities and it depended less on temporal factors (see Fig. 3a).

#### Estimation over time and number (Expt 1b)

In sharp contrast to the individuation condition, there were no significant main effects and no interactions between them for estimation performance, number: *F*(2, 34) = 2.8, *p* < .08, η_p_^2^ = 0.14; time: *F*(3, 51) = 1.6, *p* < .20, η_p_^2^ = 0.09; interaction: *F*(6, 102) = 2.1, *p* < .07, η_p_^2^ = 0.11 (see Fig. 3b). For all three number ranges (≈10, ≈20, ≈30 items), performance was at around 80% correct and stable across the different SOAs (33–200 ms). This pattern of results suggests that estimation, unlike individuation, does not depend on temporal factors. It is important to note that estimation performance was at a high level even for the shortest SOA (33 ms). Interestingly, previously we found a very similar pattern for the mere detection of a second target display after the first mask presentation (Wutz & Melcher, 2013). Thus, this suggests that stimulus detection is largely sufficient to provide the information necessary in order to approximately estimate item numbers.

#### Comparison between individuation and estimation over time (Expt 1 and b)

To statistically pin down the differences between tasks as a function of time, we collapsed over the factor item number (one to four items for individuation, ≈10–≈30 items for estimation). A two-way repeated-measures ANOVA revealed significant main effects of task, *F*(1, 17) = 7.1, *p* < .017, η_p_^2^ = 0.3, and time, *F*(3, 51) = 62.3, *p* < 0, η_p_^2^ = 0.79, as well as a significant interaction between the factors, *F*(3, 51) = 38.9, *p* < 3.2 × 10^-13^, η_p_^2^ = 0.7. Performance was slightly better in the estimation compared with the individuation task. The highly significant interaction, however, suggests that this effect was largely driven by short SOAs, which affected performance for individuation, but not for estimation. In sum, we found a strong impact of temporal factors—the mask-item(s) SOA—on individuation performance, which was completely absent for estimation (see Fig. 3). The individuation and estimation of object quantities evolves with different temporal dynamics.

#### Errors in individuation as a function of set size and SOA

**Fig. 4:**
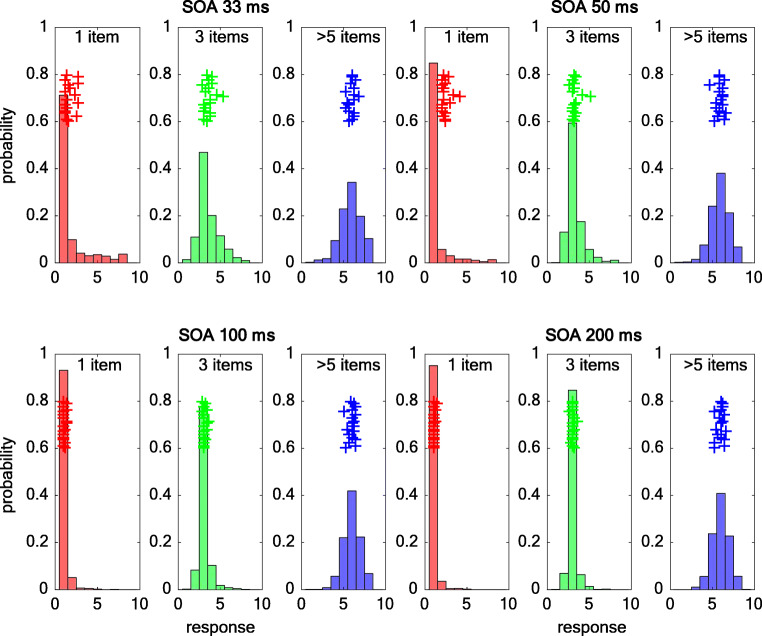
Response distributions as a function of SOA for one (red), three (green), and more than five items (blue) pooled over all participants in Expt 1a. The inset crosses show the mean responses per participant

We investigated the actually reported numbers in the individuation task as a function of set size and SOA, to more clearly distinguish between three different potential causes of the response on any given trial: (1) precise individuation, (2) approximate estimation, and (3) random guessing (see Fig. 4). When there was only a single target, participants mainly responded “1,” combined with a more scattered set of responses consistent with guessing or occasional lapses, irrespective of the SOA (*M* ± *SD* for 33-ms SOA: 1.9 ± 1.8 items; for 50-ms SOA: 1.4 ± 1.3 items; for 100-ms SOA: 1.1 ± 0.5 items; for 200-ms SOA: 1.1 ± 0.4 items). This pattern is typically found for precise individuation processes. When there were three items, however, at the short SOA values, participants often reported two or four items, and responses followed a Gaussian-like pattern around the actual value (*M* ± *SD* for 33-ms SOA: 3.6 ± 1.3 items; for 50-ms SOA: 3.3 ± 1 items). Gaussian-like response distributions are a hallmark of approximate estimation processes. A similar pattern was found with four target displays, with the Gaussian-like distribution centered around 4 and the width of the distribution being more broad for shorter SOA values (*M* ± *SD* for 33-ms SOA: 4.3 ± 1.3 items; for 50-ms SOA: 4.2 ± 1 items). Enumeration of three to four items became more precise at longer SOAs (*M* ± *SD* for three items and 100-ms SOA: 3.1 ± 0.7 items; three items and 200-ms SOA: 3 ± 0.5 items; four items and 100-ms SOA: 4.1 ± 0.7 items; four items and 200-ms SOA: 4 ± 0.7 items). In the case of larger set sizes (six to eight targets), the responses followed a relatively broad Gaussian-like distribution centered on the number 6 at all SOAs (*M* ± *SD* for 33-ms SOA: 5.9 ± 1.3 items; for 50-ms SOA: 5.9 ± 1.1 items; for 100-ms SOA: 6 ± 1 items; for 200-ms SOA: 6 ± 1 items). In other words, at very brief SOA values (33 and 55 ms), when there was more than a single item, there was evidence for an influence of estimation on responses, while for longer SOAs (100 and 200 ms) the influence of estimation on responses for set sizes of four or less appeared to be greatly diminished.

### Interim discussion

As in previous studies (Wutz et al., 2012; Wutz & Melcher, 2013; Wutz et al., 2014), we found that reaching the “normal” subitizing pattern of high percentage correct performance across the range of three or four items required more than 100 ms. As in the previous studies, there was a significant interaction between numerosity and time. To more precisely and quantitatively assess the individuation time courses for each numerosity level separately, we fitted logarithmic functions to the data and extracted the SOA-value, at which performance reached a critical threshold of 75% correct responses. This revealed that even within the subitizing range smaller numerosities reached high performance faster than the three or four item displays (SOA value for 75% correct responses for one item: 32 ms, two items: 73 ms, three items: 110 ms, 4 items: 209 ms). Moreover, enumeration was better for one item than for two items, even up to 100 ms. This pattern reflected a specific effect of SOA on performance within the subitizing range, since performance was flat for displays of six or more items.

In sharp contrast to enumeration in the subitizing range, accuracy in the numerical estimation task was not affected by the effective duration of the visual stimulus. Participants performed around 80% correct on the estimation task, irrespective of the effective duration of the stimulus. Together with the finding that enumeration outside the subitizing range was flat, these results are consistent with suggestions that estimation involves a radically different mechanism from individuation (Burr et al., 2010; Piazza et al., 2011). Even at the shortest stimulus onset asynchrony (33 ms), numerical estimation was excellent, and indeed as good as for the longest SOAs (200 ms and 500 ms). By contrast, the participants correctly enumerated/individuated that there were exactly three or four items less than 50% of the time at the 33-ms SOA.

Thus, we can estimate that 33 ms or less of effective processing time is sufficient for registering features sufficiently to support perception of approximate numerosity. This provides an estimate of the temporal resolution of numerical estimation. Our findings are consistent with the idea that the temporal resolution of ensemble processing is sufficient to allow useful and meaningful information to be extracted within 50 ms (Chong & Treisman, 2003).

A closer examination on the actual responses in the individuation task revealed distinct patterns for precise individuation and approximate estimation processes depending on the set size and the SOA. When there was only one item, performance was accurate even at the fastest SOA, and errors were relatively widely distributed across the other items as consistent with errors being largely due to guessing or lapses. For three items, the pattern of errors was consistent with responses reflecting a combination of exact enumeration and some degree of estimation depending on temporal factors (SOA). There were relatively few errors that seemed to reflect guessing. This finding is consistent with the idea that the estimation process can act across all set sizes, with the difference in error distribution in the subitizing range due to an additional process, which is the attentive individuation of each item (Burr et al., 2010). When set size exceeded the subitizing range, the pattern of responses looked similar to a Gaussian distribution centered on number 6. This distribution was similar in shape across all values of SOA, consistent with an estimation process. Overall, small set sizes (in particular, one item) showed little influence of estimation, while set size of three was consistent with a combination of estimation and exact enumeration depending on SOA, and in contrast, performance outside the subitizing range was best explained by approximate estimation, reflecting statistical or ensemble processing, largely equal across the different levels of SOA.

The pattern of responses for the six to eight item set size in the individuation task seemed consistent with an estimation process. However, it is challenging to directly compare this pattern with the 11–35-item set size since the latter (Expt 1b) involved a two-interval forced-choice procedure rather than giving an exact number response. To better compare the two tasks, we repeated the estimation task in a second experiment, in which participants responded with a specific number of target items. This allowed us to measure the pattern of responses, including errors, and compare that with those found in Experiment 1a.

## Experiment 2

### Method

#### Participants

Eighteen different participants (13 females, 16 right-handed, mean age = 25.2 years, *SD* = 5.5 years) took part in the experiment. There was no overlap with the participants of Experiment 1. All participants provided written informed consent, as approved by the institutional ethics committee. They took part in exchange for course credit or a small payment and had normal or corrected-to-normal vision.

#### Stimuli and apparatus

The stimuli and equipment were identical to those used in Experiment 1 (see Figs. 1 and 2).

#### Procedure

All subjects received verbal and written instructions about each task and completed 20 practice trials prior to the main experiment. Each trial began with a central fixation dot (black, 0.5° VA) on a white background for 500 ms. Then, the random line masking pattern was presented for a specific duration to control the stimulus onset asynchrony (SOA) between the onset of the mask and the target item(s). There were five SOAs (33, 50, 100, 200, 500 ms) and one “no-mask” control condition. Randomly assigned item quantities in three different number ranges were used. Either 11–15 (thereafter called ≈10 items), 21–25 (≈20 items), or 31–35 items (≈30 items) were shown on each trial. Unlike in Experiment 1, participants gave an exact estimate at the end of each trial rather than comparing two intervals. Thus, the response was matched to the individuation task of the first experiment (see Fig. 1). The estimation experiment comprised 5 blocks of 72 trials each. Each combination of mask-item(s) SOA (33, 50, 100 or 200) and item number (≈10, ≈20, ≈30 items) was shown six times per block in random order.

#### Data analysis

We analyzed the average error in terms of the signed difference between the reported and the presented number of items for each numerosity range (≈10, ≈20, ≈30 items) as a function of SOA with a two-way repeated-measures ANOVA. Moreover, we aimed to more directly compare the results of Experiment 1a and 2, in which exact numerical responses were reported for individuation (in Expt 1a) and estimation (in Expt 2). To this end, we calculated the accuracy coefficient (AC) and the variation coefficient (VC; Cheng et al., 2019) for the numerical judgments in each experiment (see Eqs. 1 and 2). The two measures are scaled relative to the (on average) reported number per numerosity level and thus allows for better comparability across experiments with different number ranges (one to eight items in Expt 1a; 11–35 items in Expt 2).

1 $$ \mathrm{AC}=\mathrm{Mean}\ \left(\mathrm{reported}\hbox{--} \mathrm{presented}\ \mathrm{number}\right)/\mathrm{Mean}\ \left(\mathrm{reported}\ \mathrm{number}\right) $$

2 $$ \mathrm{VC}=\mathrm{Standard}\ \mathrm{deviation}\ \left(\mathrm{reported}\ \mathrm{number}\right)/\mathrm{Mean}\ \left(\mathrm{reported}\ \mathrm{number}\right) $$

### Results

**Fig. 5:**
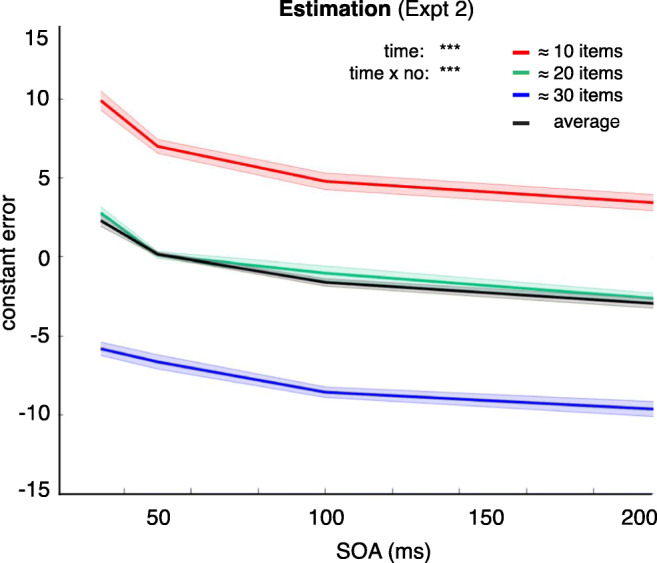
Constant error as a function of SOA for estimation responses in Experiment 2

We found a significant main effect of SOA, *F*(3, 51) = 72.1, *p* < 0, η_p_^2^ = 0.81, and a significant interaction between SOA and number range, *F*(6, 102) = 4.6, *p* < 3.9 × 10^-4^, η_p_^2^ = 0.21. As can be seen in Fig. 5, the participant responses showed a specific pattern of change over time, with an overestimation of numerosity at the shortest SOA, perhaps due to temporal integration of mask and targets, followed by an underestimation at the longest SOAs. Looking at the three different item ranges, it is also clear that there is a general regression towards the mean numerosity (which was 23, given that numerosity ranged from 11–35), which is apparent in the largely consistent error for large numerosities (around six to eight items reported less than actually presented) and for small numerosities (around six to eight items reported more than actually presented). This is consistent with previous findings showing serial dependence in numerosity estimation (Fornaciai & Park, 2018; Valsecchi, Stucchi, & Scocchia, 2018). In other words, the overall pattern is not that of a general improvement with SOA (error actually increases for 31–35 item displays), but of a general trend in underestimation, increasing with longer SOA, accompanied by an overall response bias towards the mean number of items presented across all trials (which may reflect a serial dependence effect; Fornaciai & Park, 2018, 2020).

**Fig. 6:**
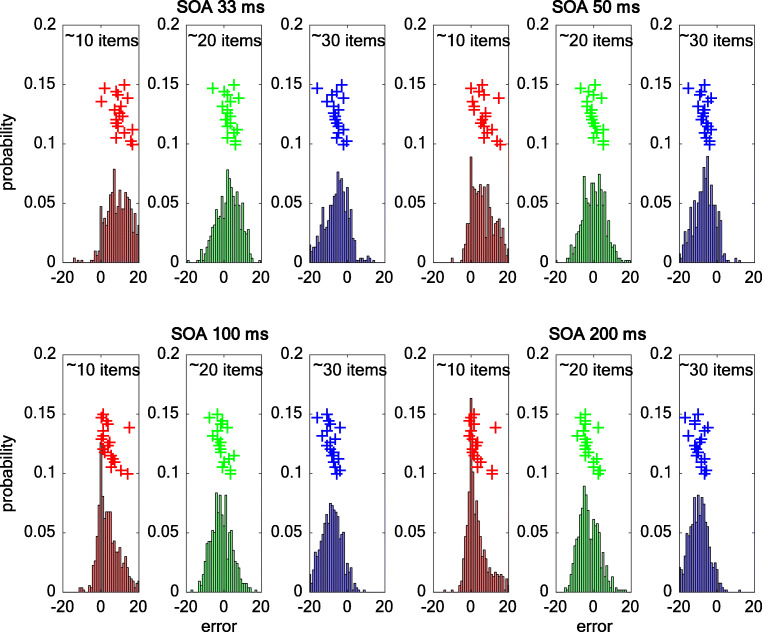
Error distributions as a function of SOA for ≈10 (red), ≈20 (green), and ≈30 items (blue) pooled over all participants in Experiment 2. The inset crosses show the mean errors per participant

The design of Experiment 2 also allowed us to directly compare the pattern of responses found for individuation (Expt 1a) with those for estimation. As shown in Fig. 5, the error pattern was largely Gaussian shaped across all number ranges and values of SOA. This pattern is similar to what had been found for six to eight item set size in Experiment 1a and for the brief (33 ms) SOA for set sizes of three or four items in the individuation task (but not for longer SOAs; see Fig. 3). The error responses also reflect the trend towards overestimation for ≈10 items and underestimation for around ≈30 items, consistent with a regression towards the mean across the estimation task (see Table 1 for descriptive statistics on the error distributions) (Fig. 6).

**Table 1:** Descriptive statistics for the error distributions

	33 ms SOA		50 ms SOA		100 ms SOA		200 ms SOA	
	*M*	*SD*	*M*	*SD*	*M*	*SD*	*M*	*SD*
~ 10 items	9.9	± 6.7	7	± 6.6	4.8	± 10.7	3.4	± 6
~ 20 items	2.8	± 10.4	0.1	± 6.1	−1	± 10.3	−2.6	± 5.6
~ 30 items	−5.8	± 6.4	−6.6	± 8.4	−8.6	± 5.7	−9.7	± 10.3

**Fig. 7:**
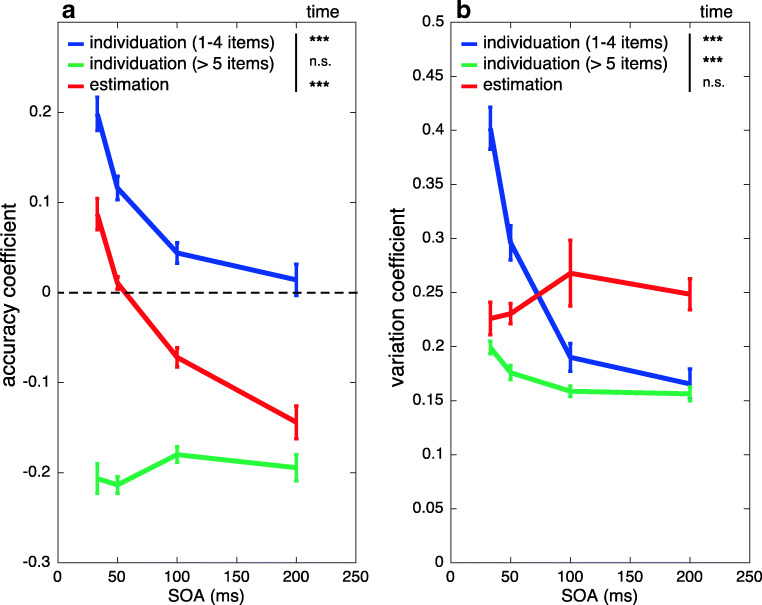
Accuracy coefficient (**a**) and variation coefficient (**b**) measures as a function of SOA for individuation of small (1–4 items) and large item numbers (>5 items) in Expt 1a and for estimation in Expt 2. For the accuracy coefficient, a difference from zero is indicative of the absence of bias, and positive/negative numbers show a tendency to either overestimate or underestimate numerosity. A lower variation coefficient indicates that the same or similar response was given across trials, whereas higher values indicate greater variability in responses

To further compare performance as a function of SOA between small and large numerosity ranges in Experiments 1a and 2, we calculated the accuracy coefficient (AC; see Method section) and variation coefficient measures (VC; see Method section). In practical terms, AC gives an estimate of accuracy or bias in overall (mean) numerical processing. Values closer to AC = 0 indicate a lack of bias, whereas positive AC values indicate overestimation and negative AC values show underestimation. As shown in Fig. 7a, for individuation within the subitizing range (pooled over one to four items), AC converged towards zero, with longer SOAs showing a strong effect of temporal factors on individuation performance, *F*(3, 51) = 28.8, *p* < 4.9 × 10^-11^, η_p_^2^ = 0.63. By contrast, individuation performance for six to eight items did not show any significant change with SOA, *F*(3, 51) = 1.4, *p* < .27, η_p_^2^ = 0.08. Importantly for estimation (pooled over 11–35 items), we also found a significant main effect of SOA on AC, *F*(3, 51) = 50.4, *p* < 2.8 × 10^-15^, η_p_^2^ = 0.75. However, this pattern of results did not show better accuracy with increasing SOAs, but instead a change in bias from overestimation to underestimation.

Low VC indicates low variability in the response such that similar answers are given from trial to trial. Thus, VC can be interpreted as a measure of relative response precision, scaled to numerosity. For exact individuation of one to four items, we found a sharp decrease in VC (indicating higher precision) as a function of SOA, *F*(3, 51) = 47.7, *p* < 8 × 10^-15^, η_p_^2^ = 0.74 (see Fig. 7b). Individuation of >5 items showed an intermediate response, with a small but statistically significant decrease in VC with longer SOAs, *F*(3, 51) = 11.1, *p* < 1 × 10^-5^, η_p_^2^ = 0.4. For estimation, however, there was no significant effect of SOA on VC, indicating that the response precision remained constant across temporal factors, *F*(3, 51) = 1, *p* < .4, η_p_^2^ = 0.06.

In this experiment, the number of items in the estimation task ranged from 11 to 35. At higher densities, it has been proposed that texture, rather than numerical estimation, may determine performance, leading to a change from Weber’s law to the square root law (for review, see Burr, Anobile, & Arrighi, 2017). We investigated this by measuring the VC as a function of set size across both experiments (from one to four and six to eight in Experiment 1a; ~10, ~20, ~30 items in Experiment 2), at the longest SOA (200 ms). According to Weber’s law, the variability in responses should increase in proportion to the change in numerosity, leading to a relatively constant value of the Weber’s fraction (and VC) across numerosity values (for review, see Burr et al., 2017). In contrast, if increased numerosity past a certain critical point leads to a switch towards texture-based processing, then variability should increase according to the square root law rather than staying a constant Weber fraction (for review, see Burr et al., 2017). We found that the VC at the longest SOA (200 ms) was relatively flat, with no significant effect of set size, for both Experiment 1a, *F*(4, 68) = 1.5, *p* < .23, η_p_^2^ = 0.08, and Experiment 2, *F*(2, 34) = 0.5, *p* < .64, η_p_^2^ = 0.03 . This pattern matches what has been reported previously in number perception tasks within the estimation range (for review, see Burr et al., 2017).

## General discussion

Across two experiments, we investigated the way in which limiting the effective processing time of a group of items influenced numerosity judgments. Previous work has suggested that the temporal resolution of ensemble processing is quite high, on the order of 50 ms (Chong & Treisman, 2003). However, there is evidence for a longer temporal integration window, in which judgments can become more precise with longer display times (Whiting & Oriet, 2011). It has also been argued that the time frame of ensemble perception may depend on the features and/or task in question (Hubert-Wallander & Boynton, 2015). Here, we investigated the time frame of ensemble processing of numerosity, one of the most fundamental statistical properties of a set of items.

To closely investigate the evolution of estimation over effective processing time, we took advantage of a combined forward plus simultaneous masking technique, which has been shown to be effective for characterizing the temporal evolution of individuating small numbers of items (Wutz et al., 2012; Wutz et al., 2014). This also allowed us to directly compare numerosity judgments in the subitizing range with performance for six to eight or 11 to 35 items. It remains a matter of debate whether and how ensemble processing is similar across different ranges of items. For example, is estimating the average size of two items similar, in terms of mechanisms, to estimating the average size of 20 items?

The first main finding from this study is that the time course of numerosity judgments, even within a range of one to eight items, differs as a function of the number of target items. Accuracy, as measured by the ability to give the correct exact numerosity, remained low whenever set size exceeded around four items across the SOA values from 33–200 ms. Within the one to four item subitizing range, accuracy improved with longer SOAs, first for the one-item display and eventually also for three or four targets. This replicates previous results with small numbers of items (Wutz et al., 2012; Wutz & Melcher, 2013; Wutz et al., 2014). In terms of temporal resolution, it is clear that participants needed 100 ms or more to reach good performance in the subitizing range. Outside of the subitizing range, the pattern of errors was consistent with an estimation mechanism, and performance did not significantly improve for longer SOAs.

An interesting middle case is Set Size 3, where the pattern of errors seemed to match what would be expected if responses reflected a combination of estimation (extracted within about 33 or 50 ms) and exact individuation (improving over more than 100 ms). Our findings agree with the proposal that the estimation process is active across all set sizes (Burr et al., 2010). The fact that within the subitizing range there is a difference in error distribution has been suggested to be due to the attentive selection of individual items for a small number of stimuli (Burr et al., 2010). Thus, for three to four items, the used strategy for numerical processing (estimation or individuation) depends on the effective duration of the stimulus (i.e., its integration window).

In terms of underlying mechanisms, the repeated finding of an advantage, in terms of accuracy and precision, for small numerosities (one to four) items, has been linked to object individuation mechanisms, which can operate for such small set sizes. In particular, the object enumeration process has been linked to selective attention to a small number of items. Reducing the ability to rapidly focus attention on the items increases variability and makes both behavioral (Burr et al., 2010) and neural (Hyde & Wood, 2011) responses for small set sizes more similar to those found for estimation. In the current study, we found that very brief values of SOA also led to responses that were more like estimation. These results are consistent with a limited temporal window to attend to and individuate objects (Wutz et al., 2012; Wutz & Melcher, 2013). Without sufficient time to operate, selective spatial attention is not able to individuate each item as a unique entity, meaning that only a less precise estimate of numerosity is possible.

A key finding in the present study is that the time course of estimation for larger numerosities (outside the subitizing range), as measured in two experiments using different response tasks, was quite different from the time course of individuation within the subitizing range. In the first experiment, using a two-interval comparison task, we found that accuracy did not differ as a function of SOA. In the second experiment, participants reported the exact numerosity in order to more directly match the individuation task in the first experiment. In contrast with the individuation task, there was not a general increase in performance as a function of SOA. Errors actually increased with longer SOAs for 31–35 item displays. Instead, there were two main patterns in the results. First, the reported number became smaller for longer SOAs, as shown by the downward slope (towards negative errors) over time. The second main pattern in Experiment 2 was a tendency of responses to regress to the mean number of items (23 targets), such that a smaller number of stimuli (≈10 items) was overestimated, and a larger number of stimuli (≈30 items) was underestimated. Critically, neither of these patterns was apparent in the first experiment for the small number individuation task (one to four items).

The overall pattern of results is consistent with theories that the underlying mechanisms allowing for estimation are different from those of small set-size individuation (Burr et al., 2010; Piazza et al., 2011). Here, we demonstrate differences in the time course of the two mechanisms. We can infer that even 33-50 ms is sufficient for registering visual features to a point that is sufficient to support perception of approximate numerosity. This provides an estimate of the temporal resolution of numerical estimation. Our findings are consistent with the idea that the temporal resolution of ensemble processing is sufficient to allow for some useful and meaningful information to be extracted within 50 ms (Chong & Treisman, 2003). We also found further changes within a temporal window of 200 ms, but it was difficult to distinguish between purely perceptual and decision-making effects, given that both over/underestimation and regression to the mean across trials may be related to decision strategies or other factors. Most previous studies of estimation have used comparison (as in Experiment 1b), meaning that there are fewer studies in the literature that would be able to find such effects. Other reports have shown that numerosity can be underestimated when items are enclosed, connected or clustered together (He, Zhang, Zhou, & Chen, 2009; He, Zhou, Zhou, He, & Chen, 2015; Im, Zhong, & Halberda, 2016). The presence of the simultaneous mask may have induced such grouping effects. Future work is necessary to distinguish between more purely perceptual and more decision-making accounts of these two patterns in the estimation data.

Overall, the current findings are consistent with the claim that ensemble processing, at least in the case of the numerosity of the set, is as fast as processing a single item. In fact, it could be considered faster in the sense that individuation of three or four items improves over a longer time period, whereas no such improvement was found in the estimation of multiple items. Moreover, in the case of small sets of around three to four items, the results are consistent with the use of a combination of approximate estimation for short SOAs and exact enumeration for longer SOAs. Though the current findings provide further evidence for rapid temporal resolution of ensemble processing, it is of course important to remember that the time course may vary for different features or tasks (Hubert-Wallander & Boynton, 2015).

It is interesting to compare the time course found here, for simultaneous presentation of stimuli, to studies using sequential presentations of items (Anobile, Arrighi, & Burr, 2019; Cheng et al., 2019). In a recent study, Cheng et al. (2019) varied the rate of presentation of items, with the processing window of each item (before it was replaced by another item) ranging from 100–400 ms (100, 300, and 400 ms were tested). When items were presented every 100 ms, participants made a high number of errors, even within the subitizing range. The next fastest rate tested in that study, 300 ms, yielded performance similar to that found with simultaneous presentation of the items, with low error rates for one to three items. Consistent with many studies using simultaneous presentation, errors increased only once set sizes exceeded around four to five items. In all of their conditions with 300 ms or more per item, the individuation process could be completed before a new item arrived, leading to typical patterns of subitizing. In contrast, with only 100 ms, the individuation process (and updating of numerical information in memory) would presumably not yet be finished by the time a new item arrived, leading to errors even within the subitizing range. The current findings are compatible with those of Cheng et al. (2019), who investigated sequential presentation paradigms for individuation and observed a more flexible allocation of resources. The relatively fixed temporal window for object individuation found here could serve as the basic building block for enumeration operations. In this way, individuation in sequential paradigms may depend on a series of relatively fixed temporal windows needed to flexibly allocate resources for working memory and the updating of numerosity over longer time periods.

In summary, we replicated the finding that exact numerosity judgments, which require object individuation, improved within the subitizing range over a period of 100–200 ms. By contrast, numerical estimation, which can be supported by ensemble processing, was found to be largely independent of the effective duration of the stimulus in Experiment 1b. The second experiment showed that there was an increase in underestimation with longer effective stimulus durations, combined with regression towards the mean, but this did not improve performance in general. Overall, this pattern of results suggests that ensemble processing of “the forest” can occur as fast as, if not faster than, object-specific processing of “the trees” as individuals.
